# 3-(Phenyl­imino)indolin-2-one

**DOI:** 10.1107/S160053681000259X

**Published:** 2010-01-27

**Authors:** Abdusalam Al Subari, Rachid Bouhfid, Hafid Zouihri, El Mokhtar Essassi, Seik Weng Ng

**Affiliations:** a Laboratoire de Chimie Organique Hétérocyclique, Pôle de Compétences Pharmacochimie, Université Mohammed V-Agdal, BP 1014 Avenue Ibn Batout, Rabat, Morocco; b Institute of Nanomaterials and Nanotechnology, Avenue de l’Armée Royale, Madinat El Irfane, 10100 Rabat, Morocco; c CNRST Division of UATRS Angle Allal Fassi/FAR, BP 8027 Hay Riad, 10000 Rabat, Morocco; d Department of Chemistry, University of Malaya, 50603 Kuala Lumpur, Malaysia

## Abstract

The imino C=N double bond in the title compound, C_14_H_10_N_2_O, exists in an *E* conformation, with the phenyl ring being twisted by 80.7 (1)° in one independent mol­ecule and by 81.4 (1)° in the other with respect to the plane of the indoline fused-ring system. The two independent mol­ecules are linked by N—H⋯O hydrogen bonds, forming a zigzag chain running along the *a* axis.

## Related literature

For the synthesis, see: Grimshaw & Begley (1974 ▶). For the crystal structures of other phenyl-substituted derivatives, see: Akkurt *et al.* (2003 ▶); Hökelek *et al.* (2006 ▶); Öztürk *et al.* (2003 ▶).
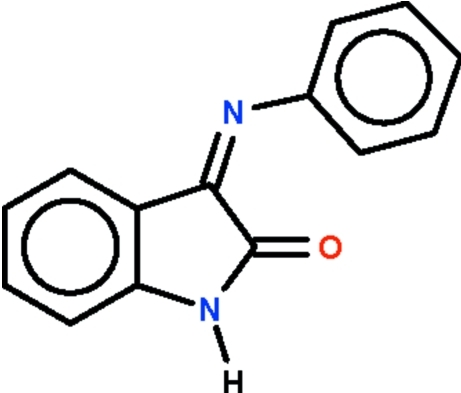


## Experimental

### 

#### Crystal data

C_14_H_10_N_2_O
*M* *_r_* = 222.24
Orthorhombic, 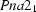

*a* = 20.1647 (4) Å
*b* = 5.0223 (1) Å
*c* = 21.8791 (5) Å
*V* = 2215.77 (8) Å^3^
*Z* = 8
Mo *K*α radiation
μ = 0.09 mm^−1^
*T* = 293 K
0.3 × 0.3 × 0.3 mm

#### Data collection

Bruker APEXII diffractometer
15055 measured reflections
2619 independent reflections
2042 reflections with *I* > 2σ(*I*)
*R*_int_ = 0.030

#### Refinement

*R*[*F* ^2^ > 2σ(*F* ^2^)] = 0.034
*wR*(*F* ^2^) = 0.118
*S* = 1.10
2619 reflections
315 parameters
3 restraints
H atoms treated by a mixture of independent and constrained refinement
Δρ_max_ = 0.19 e Å^−3^
Δρ_min_ = −0.20 e Å^−3^

### 

Data collection: *APEX2* (Bruker, 2005 ▶); cell refinement: *SAINT* (Bruker, 2005 ▶); data reduction: *SAINT*; program(s) used to solve structure: *SHELXS97* (Sheldrick, 2008 ▶); program(s) used to refine structure: *SHELXL97* (Sheldrick, 2008 ▶); molecular graphics: *X-SEED* (Barbour, 2001 ▶); software used to prepare material for publication: *publCIF* (Westrip, 2010 ▶).

## Supplementary Material

Crystal structure: contains datablocks global, I. DOI: 10.1107/S160053681000259X/bt5177sup1.cif

Structure factors: contains datablocks I. DOI: 10.1107/S160053681000259X/bt5177Isup2.hkl

Additional supplementary materials:  crystallographic information; 3D view; checkCIF report

## Figures and Tables

**Table 1:** Hydrogen-bond geometry (Å, °)

*D*—H⋯*A*	*D*—H	H⋯*A*	*D*⋯*A*	*D*—H⋯*A*
N1—H1⋯N4^i^	0.86 (3)	2.11 (2)	2.940 (3)	161 (3)
N3—H3⋯N2	0.86 (3)	2.28 (3)	3.111 (3)	164 (3)

Symmetry code: (i) 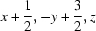.

**Fig. 1.**
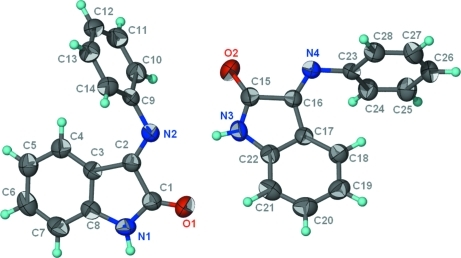
Anisotropic displacement ellipsoid plot (Barbour, 2001) of the two independent molecules of C14H10N2O at the 50% probability level; hydrogen atoms are drawn as spheres of an arbitrary radius.

## supplementary crystallographic information

### Experimental

The compound was synthesized from the reaction of isatin and aniline in ethanol according to a literature method (Grimshaw & Begley, 1974), and crystals were obtained by recrystallization from ethanol.

### Refinement

Carbon-bound H-atoms were placed in calculated positions (C—H 0.93 Å) and were included in the refinement in the riding model approximation, with *U*(H) set to 1.2*U*(C). The amino H-atoms were located in a difference Fourier map, and were refined isotropically with a distance restraint of N–H 0.86±0.01 Å. Friedel pairs were merged.

### Crystal data

**Table:**

C_14_H_10_N_2_O	*F*(000) = 928
*M**_r_* = 222.24	*D*_x_ = 1.332 Mg m^−^^3^
Orthorhombic, *P**n**a*2_1_	Mo *K*α radiation, λ = 0.71073 Å
Hall symbol: P 2c -2n	Cell parameters from 3521 reflections
*a* = 20.1647 (4) Å	θ = 2.7–25.7°
*b* = 5.0223 (1) Å	µ = 0.09 mm^−^^1^
*c* = 21.8791 (5) Å	*T* = 293 K
*V* = 2215.77 (8) Å^3^	Block, orange
*Z* = 8	0.3 × 0.3 × 0.3 mm

### Data collection

**Table:**

Bruker APEXII diffractometer	2042 reflections with *I* > 2σ(*I*)
Radiation source: fine-focus sealed tube	*R*_int_ = 0.030
graphite	θ_max_ = 27.5°, θ_min_ = 1.9°
φ and ω scans	*h* = −24→26
15055 measured reflections	*k* = −3→6
2619 independent reflections	*l* = −28→28

### Refinement

**Table:**

Refinement on *F*^2^	Primary atom site location: structure-invariant direct methods
Least-squares matrix: full	Secondary atom site location: difference Fourier map
*R*[*F*^2^ > 2σ(*F*^2^)] = 0.034	Hydrogen site location: inferred from neighbouring sites
*wR*(*F*^2^) = 0.118	H atoms treated by a mixture of independent and constrained refinement
*S* = 1.10	*w* = 1/[σ^2^(*F*_o_^2^) + (0.0745*P*)^2^ + 0.0171*P*] where *P* = (*F*_o_^2^ + 2*F*_c_^2^)/3
2619 reflections	(Δ/σ)_max_ = 0.001
315 parameters	Δρ_max_ = 0.19 e Å^−^^3^
3 restraints	Δρ_min_ = −0.20 e Å^−^^3^

### Fractional atomic coordinates and isotropic or equivalent isotropic displacement parameters (Å^2^)

**Table:**

	*x*	*y*	*z*	*U*_iso_*/*U*_eq_	
O1	0.36628 (12)	0.9072 (6)	0.49888 (10)	0.0769 (7)	
O2	0.11993 (12)	0.6636 (6)	0.52675 (12)	0.0816 (8)	
N1	0.44843 (11)	0.6264 (6)	0.53285 (11)	0.0543 (6)	
N2	0.28865 (11)	0.6607 (5)	0.59271 (10)	0.0505 (6)	
N3	0.20427 (11)	0.9352 (5)	0.49173 (10)	0.0535 (6)	
N4	0.04413 (11)	0.9133 (5)	0.43186 (11)	0.0525 (6)	
C1	0.38742 (14)	0.7371 (6)	0.53269 (13)	0.0535 (7)	
C2	0.34907 (12)	0.5996 (5)	0.58405 (11)	0.0462 (6)	
C3	0.39537 (12)	0.4079 (5)	0.61115 (11)	0.0446 (6)	
C4	0.39058 (14)	0.2185 (6)	0.65685 (13)	0.0519 (7)	
H4	0.3517	0.2014	0.6794	0.062*	
C5	0.44415 (16)	0.0553 (6)	0.66857 (15)	0.0605 (7)	
H5	0.4414	−0.0724	0.6993	0.073*	
C6	0.50158 (16)	0.0802 (7)	0.63515 (17)	0.0663 (8)	
H6	0.5370	−0.0327	0.6436	0.080*	
C7	0.50835 (15)	0.2678 (7)	0.58932 (15)	0.0609 (8)	
H7	0.5475	0.2840	0.5672	0.073*	
C8	0.45450 (13)	0.4302 (6)	0.57769 (12)	0.0481 (6)	
C9	0.25376 (12)	0.5309 (5)	0.64115 (12)	0.0454 (6)	
C10	0.20928 (13)	0.3331 (7)	0.62764 (14)	0.0565 (7)	
H10	0.2017	0.2850	0.5872	0.068*	
C11	0.17563 (15)	0.2051 (7)	0.67433 (17)	0.0619 (8)	
H11	0.1464	0.0673	0.6653	0.074*	
C12	0.18528 (14)	0.2810 (7)	0.73385 (15)	0.0594 (8)	
H12	0.1628	0.1938	0.7651	0.071*	
C13	0.22774 (15)	0.4843 (6)	0.74728 (13)	0.0565 (7)	
H13	0.2334	0.5381	0.7876	0.068*	
C14	0.26251 (16)	0.6108 (6)	0.70102 (13)	0.0536 (7)	
H14	0.2916	0.7488	0.7102	0.064*	
C15	0.14226 (13)	0.8292 (7)	0.49218 (13)	0.0539 (7)	
C16	0.10494 (12)	0.9666 (5)	0.44047 (11)	0.0455 (6)	
C17	0.15210 (13)	1.1524 (5)	0.41286 (11)	0.0437 (6)	
C18	0.14711 (14)	1.3414 (6)	0.36677 (13)	0.0528 (7)	
H18	0.1080	1.3596	0.3447	0.063*	
C19	0.20099 (16)	1.5019 (6)	0.35414 (15)	0.0609 (7)	
H19	0.1983	1.6295	0.3235	0.073*	
C20	0.25942 (17)	1.4720 (7)	0.38751 (17)	0.0661 (8)	
H20	0.2955	1.5803	0.3785	0.079*	
C21	0.26500 (16)	1.2858 (7)	0.43355 (16)	0.0620 (8)	
H21	0.3042	1.2679	0.4555	0.074*	
C22	0.21093 (13)	1.1269 (5)	0.44624 (12)	0.0472 (6)	
C23	0.00927 (12)	1.0342 (6)	0.38269 (12)	0.0461 (6)	
C24	0.01763 (15)	0.9423 (6)	0.32334 (14)	0.0576 (7)	
H24	0.0478	0.8065	0.3154	0.069*	
C25	−0.01848 (16)	1.0510 (7)	0.27619 (16)	0.0610 (8)	
H25	−0.0126	0.9887	0.2365	0.073*	
C26	−0.06293 (15)	1.2504 (7)	0.28746 (16)	0.0630 (8)	
H26	−0.0869	1.3255	0.2555	0.076*	
C27	−0.07213 (15)	1.3401 (7)	0.34652 (17)	0.0687 (9)	
H27	−0.1025	1.4755	0.3541	0.082*	
C28	−0.03689 (14)	1.2315 (7)	0.39430 (14)	0.0586 (7)	
H28	−0.0441	1.2903	0.4341	0.070*	
H1	0.4791 (10)	0.653 (6)	0.5061 (10)	0.052 (8)*	
H3	0.2338 (12)	0.883 (7)	0.5172 (12)	0.064 (9)*	

### Atomic displacement parameters (Å^2^)

**Table:**

	*U*^11^	*U*^22^	*U*^33^	*U*^12^	*U*^13^	*U*^23^
O1	0.0687 (14)	0.0941 (18)	0.0678 (15)	0.0084 (12)	0.0068 (11)	0.0340 (13)
O2	0.0684 (14)	0.104 (2)	0.0721 (14)	−0.0040 (13)	−0.0001 (12)	0.0422 (15)
N1	0.0457 (13)	0.0711 (16)	0.0461 (12)	−0.0002 (11)	0.0104 (10)	0.0017 (12)
N2	0.0423 (12)	0.0625 (15)	0.0468 (12)	0.0047 (10)	0.0007 (9)	0.0070 (11)
N3	0.0483 (12)	0.0653 (15)	0.0470 (12)	0.0081 (11)	−0.0064 (10)	0.0028 (11)
N4	0.0419 (12)	0.0659 (15)	0.0498 (12)	0.0040 (11)	0.0037 (9)	0.0097 (11)
C1	0.0477 (15)	0.0673 (19)	0.0454 (15)	−0.0002 (13)	0.0020 (12)	0.0021 (14)
C2	0.0440 (13)	0.0533 (15)	0.0414 (12)	−0.0015 (11)	−0.0017 (10)	−0.0023 (12)
C3	0.0424 (13)	0.0466 (14)	0.0447 (13)	0.0000 (11)	−0.0018 (10)	−0.0070 (11)
C4	0.0528 (15)	0.0507 (15)	0.0523 (16)	−0.0015 (12)	−0.0007 (12)	−0.0013 (13)
C5	0.0673 (18)	0.0514 (16)	0.0628 (17)	0.0045 (14)	−0.0089 (15)	0.0015 (14)
C6	0.0586 (17)	0.0610 (18)	0.079 (2)	0.0137 (15)	−0.0129 (15)	−0.0068 (17)
C7	0.0498 (16)	0.0683 (19)	0.0645 (19)	0.0081 (13)	0.0051 (14)	−0.0094 (16)
C8	0.0452 (14)	0.0521 (15)	0.0471 (13)	0.0008 (11)	0.0006 (11)	−0.0098 (12)
C9	0.0355 (12)	0.0527 (15)	0.0479 (13)	0.0082 (11)	0.0022 (10)	0.0033 (12)
C10	0.0534 (16)	0.0620 (18)	0.0541 (16)	−0.0013 (14)	−0.0060 (14)	−0.0022 (14)
C11	0.0501 (16)	0.0592 (17)	0.076 (2)	−0.0087 (14)	−0.0041 (15)	0.0083 (16)
C12	0.0505 (17)	0.0612 (18)	0.067 (2)	0.0046 (14)	0.0100 (14)	0.0159 (15)
C13	0.0664 (17)	0.0566 (16)	0.0465 (14)	0.0090 (14)	0.0075 (13)	−0.0021 (13)
C14	0.0539 (16)	0.0525 (16)	0.0544 (16)	−0.0026 (13)	0.0024 (12)	−0.0016 (12)
C15	0.0486 (15)	0.0687 (19)	0.0444 (15)	0.0099 (14)	0.0045 (12)	0.0066 (13)
C16	0.0411 (13)	0.0532 (15)	0.0422 (13)	0.0076 (11)	0.0045 (10)	0.0025 (11)
C17	0.0444 (14)	0.0452 (14)	0.0414 (13)	0.0083 (11)	0.0055 (10)	−0.0025 (10)
C18	0.0525 (16)	0.0513 (15)	0.0545 (17)	0.0073 (12)	0.0051 (13)	0.0036 (13)
C19	0.0660 (18)	0.0527 (16)	0.0640 (17)	0.0002 (14)	0.0109 (15)	0.0083 (15)
C20	0.0609 (17)	0.0572 (18)	0.080 (2)	−0.0122 (15)	0.0092 (17)	−0.0001 (17)
C21	0.0492 (16)	0.0621 (18)	0.075 (2)	−0.0016 (13)	−0.0026 (15)	−0.0090 (17)
C22	0.0468 (14)	0.0480 (14)	0.0467 (13)	0.0062 (11)	0.0026 (11)	−0.0062 (12)
C23	0.0355 (12)	0.0510 (15)	0.0519 (14)	0.0011 (11)	0.0023 (10)	0.0069 (12)
C24	0.0606 (17)	0.0549 (17)	0.0572 (16)	0.0146 (13)	0.0025 (14)	0.0000 (14)
C25	0.0688 (19)	0.0606 (18)	0.0536 (15)	0.0011 (15)	−0.0094 (14)	−0.0036 (15)
C26	0.0542 (18)	0.0700 (19)	0.065 (2)	0.0061 (15)	−0.0105 (14)	0.0155 (16)
C27	0.0550 (18)	0.069 (2)	0.082 (2)	0.0236 (15)	0.0052 (16)	0.0144 (18)
C28	0.0542 (16)	0.0647 (19)	0.0569 (17)	0.0108 (14)	0.0077 (14)	0.0011 (14)

### Geometric parameters (Å, °)

**Table:**

O1—C1	1.208 (4)	C11—H11	0.9300
O2—C15	1.211 (4)	C12—C13	1.364 (5)
N1—C1	1.350 (4)	C12—H12	0.9300
N1—C8	1.396 (4)	C13—C14	1.386 (4)
N1—H1	0.86 (1)	C13—H13	0.9300
N2—C2	1.271 (3)	C14—H14	0.9300
N2—C9	1.429 (3)	C15—C16	1.524 (4)
N3—C15	1.359 (4)	C16—C17	1.463 (4)
N3—C22	1.391 (4)	C17—C18	1.389 (4)
N3—H3	0.86 (1)	C17—C22	1.399 (4)
N4—C16	1.269 (3)	C18—C19	1.381 (4)
N4—C23	1.421 (3)	C18—H18	0.9300
C1—C2	1.529 (4)	C19—C20	1.394 (5)
C2—C3	1.466 (4)	C19—H19	0.9300
C3—C4	1.383 (4)	C20—C21	1.379 (5)
C3—C8	1.404 (4)	C20—H20	0.9300
C4—C5	1.380 (4)	C21—C22	1.380 (4)
C4—H4	0.9300	C21—H21	0.9300
C5—C6	1.375 (5)	C23—C28	1.383 (4)
C5—H5	0.9300	C23—C24	1.389 (4)
C6—C7	1.383 (5)	C24—C25	1.376 (4)
C6—H6	0.9300	C24—H24	0.9300
C7—C8	1.382 (4)	C25—C26	1.366 (4)
C7—H7	0.9300	C25—H25	0.9300
C9—C10	1.371 (4)	C26—C27	1.381 (5)
C9—C14	1.381 (4)	C26—H26	0.9300
C10—C11	1.385 (4)	C27—C28	1.377 (4)
C10—H10	0.9300	C27—H27	0.9300
C11—C12	1.371 (5)	C28—H28	0.9300
			
C1—N1—C8	111.9 (2)	C14—C13—H13	119.9
C1—N1—H1	126 (2)	C9—C14—C13	119.7 (3)
C8—N1—H1	121 (2)	C9—C14—H14	120.2
C2—N2—C9	118.2 (2)	C13—C14—H14	120.2
C15—N3—C22	111.4 (2)	O2—C15—N3	128.0 (3)
C15—N3—H3	121 (2)	O2—C15—C16	126.2 (3)
C22—N3—H3	128 (2)	N3—C15—C16	105.7 (2)
C16—N4—C23	120.0 (2)	N4—C16—C17	134.5 (2)
O1—C1—N1	127.9 (3)	N4—C16—C15	119.4 (2)
O1—C1—C2	126.2 (3)	C17—C16—C15	105.9 (2)
N1—C1—C2	105.8 (2)	C18—C17—C22	120.2 (3)
N2—C2—C3	135.1 (3)	C18—C17—C16	133.5 (3)
N2—C2—C1	119.0 (2)	C22—C17—C16	106.1 (2)
C3—C2—C1	105.8 (2)	C19—C18—C17	119.2 (3)
C4—C3—C8	119.4 (3)	C19—C18—H18	120.4
C4—C3—C2	134.4 (2)	C17—C18—H18	120.4
C8—C3—C2	106.1 (2)	C18—C19—C20	119.8 (3)
C5—C4—C3	119.2 (3)	C18—C19—H19	120.1
C5—C4—H4	120.4	C20—C19—H19	120.1
C3—C4—H4	120.4	C21—C20—C19	121.6 (3)
C6—C5—C4	120.4 (3)	C21—C20—H20	119.2
C6—C5—H5	119.8	C19—C20—H20	119.2
C4—C5—H5	119.8	C20—C21—C22	118.3 (3)
C5—C6—C7	122.0 (3)	C20—C21—H21	120.8
C5—C6—H6	119.0	C22—C21—H21	120.8
C7—C6—H6	119.0	C21—C22—N3	128.4 (3)
C8—C7—C6	117.3 (3)	C21—C22—C17	120.8 (3)
C8—C7—H7	121.4	N3—C22—C17	110.8 (2)
C6—C7—H7	121.4	C28—C23—C24	119.5 (3)
C7—C8—N1	128.0 (3)	C28—C23—N4	120.0 (3)
C7—C8—C3	121.6 (3)	C24—C23—N4	120.4 (3)
N1—C8—C3	110.4 (2)	C25—C24—C23	120.3 (3)
C10—C9—C14	119.9 (3)	C25—C24—H24	119.8
C10—C9—N2	119.5 (3)	C23—C24—H24	119.8
C14—C9—N2	120.5 (3)	C26—C25—C24	120.2 (3)
C9—C10—C11	119.9 (3)	C26—C25—H25	119.9
C9—C10—H10	120.1	C24—C25—H25	119.9
C11—C10—H10	120.1	C25—C26—C27	119.7 (3)
C12—C11—C10	120.1 (3)	C25—C26—H26	120.1
C12—C11—H11	119.9	C27—C26—H26	120.1
C10—C11—H11	119.9	C28—C27—C26	120.8 (3)
C13—C12—C11	120.1 (3)	C28—C27—H27	119.6
C13—C12—H12	119.9	C26—C27—H27	119.6
C11—C12—H12	119.9	C27—C28—C23	119.5 (3)
C12—C13—C14	120.2 (3)	C27—C28—H28	120.3
C12—C13—H13	119.9	C23—C28—H28	120.3
			
C8—N1—C1—O1	−178.4 (3)	C22—N3—C15—O2	−178.1 (3)
C8—N1—C1—C2	0.7 (3)	C22—N3—C15—C16	0.7 (3)
C9—N2—C2—C3	3.4 (5)	C23—N4—C16—C17	5.7 (5)
C9—N2—C2—C1	−179.7 (2)	C23—N4—C16—C15	−178.2 (2)
O1—C1—C2—N2	1.5 (5)	O2—C15—C16—N4	1.7 (5)
N1—C1—C2—N2	−177.6 (3)	N3—C15—C16—N4	−177.1 (3)
O1—C1—C2—C3	179.3 (3)	O2—C15—C16—C17	178.9 (3)
N1—C1—C2—C3	0.1 (3)	N3—C15—C16—C17	0.1 (3)
N2—C2—C3—C4	0.2 (6)	N4—C16—C17—C18	0.4 (5)
C1—C2—C3—C4	−177.1 (3)	C15—C16—C17—C18	−176.1 (3)
N2—C2—C3—C8	176.3 (3)	N4—C16—C17—C22	175.8 (3)
C1—C2—C3—C8	−0.9 (3)	C15—C16—C17—C22	−0.8 (3)
C8—C3—C4—C5	0.0 (4)	C22—C17—C18—C19	0.4 (4)
C2—C3—C4—C5	175.7 (3)	C16—C17—C18—C19	175.2 (3)
C3—C4—C5—C6	−0.1 (4)	C17—C18—C19—C20	0.1 (4)
C4—C5—C6—C7	0.4 (5)	C18—C19—C20—C21	−0.4 (5)
C5—C6—C7—C8	−0.6 (5)	C19—C20—C21—C22	0.0 (5)
C6—C7—C8—N1	−177.6 (3)	C20—C21—C22—N3	−177.2 (3)
C6—C7—C8—C3	0.4 (4)	C20—C21—C22—C17	0.6 (4)
C1—N1—C8—C7	176.8 (3)	C15—N3—C22—C21	176.7 (3)
C1—N1—C8—C3	−1.4 (3)	C15—N3—C22—C17	−1.2 (3)
C4—C3—C8—C7	−0.1 (4)	C18—C17—C22—C21	−0.8 (4)
C2—C3—C8—C7	−177.0 (3)	C16—C17—C22—C21	−176.9 (2)
C4—C3—C8—N1	178.2 (2)	C18—C17—C22—N3	177.3 (2)
C2—C3—C8—N1	1.4 (3)	C16—C17—C22—N3	1.2 (3)
C2—N2—C9—C10	−104.2 (3)	C16—N4—C23—C28	−107.2 (3)
C2—N2—C9—C14	78.5 (4)	C16—N4—C23—C24	77.5 (4)
C14—C9—C10—C11	−3.1 (4)	C28—C23—C24—C25	1.6 (5)
N2—C9—C10—C11	179.5 (2)	N4—C23—C24—C25	176.9 (3)
C9—C10—C11—C12	1.9 (4)	C23—C24—C25—C26	−0.1 (5)
C10—C11—C12—C13	0.5 (5)	C24—C25—C26—C27	−0.9 (5)
C11—C12—C13—C14	−1.5 (5)	C25—C26—C27—C28	0.2 (5)
C10—C9—C14—C13	2.1 (4)	C26—C27—C28—C23	1.4 (5)
N2—C9—C14—C13	179.4 (2)	C24—C23—C28—C27	−2.3 (5)
C12—C13—C14—C9	0.3 (4)	N4—C23—C28—C27	−177.6 (3)

### Hydrogen-bond geometry (Å, °)

**Table:**

*D*—H···*A*	*D*—H	H···*A*	*D*···*A*	*D*—H···*A*
N1—H1···N4^i^	0.86 (3)	2.11 (2)	2.940 (3)	161 (3)
N3—H3···N2	0.86 (3)	2.28 (3)	3.111 (3)	164 (3)

Symmetry codes: (i) *x*+1/2, −*y*+3/2, *z*.
